# Valedictory Address to the Graduating Class of the Baltimore College of Dental Surgery, Delivered February 25th, 1858

**Published:** 1858-04

**Authors:** A. Snowden Piggot


### AMERICAN JOURNAL

OF

### DENTAL SCIENCE.

Vol. VIII. NEW SERIES—APRIL, 1858. No. 2.

### ORIGINAL COMMUNICATIONS.

### ARTICLE I.

*Valedictory Address to the Graduating Class of the Baltimore College of Dental Surgery, delivered February 25th,* 1858. By A. Snowden Piggot, M. D.

I occupy to-night a novel position before you. I am here to deliver, at one and the same time, a salutatory and a valedictory lecture. For the first time I appear before you in public, as an authorized teacher, and my only duty is to bid you farewell. The statement of the case seems to involve a contradiction in terms, and yet, there is some appropriateness in this juxtaposition of the *salve* and the *vale*.

At every well defined stage of the journey of life, it is natural to indulge in retrospect upon the past, and to peer into the distant and shadowy future. As the traveler, who has had for hours in full view some commanding eminence lying just upon his road, when he finally attains it, halts, and looks back upon the route he has just traveled, marking with his eye the different points he has passed, and then looks forward towards his ultimate destination, dim in the remote distance; so we all are prone to select certain halting spots in life, which serve us the purpose of estimating our progress and determining how far we have yet to go before we reach the goal we have in view. They, in their turn, in after life, serve as landmarks to indicate our career, and as the evening comes on, their summits, illumined by the old sunlight of hope, which is gradually sinking lower and lower under the horizon, still shed a fading glory through the gathering gloom, till the inevitable night blots them all out forever. It is the hope that fills these golden moments in early life which gives them such a durable lustre, such lasting beauty. To the young heart every thing is charming. The desert itself looks glorious in the morning. But as the evening draws on, the weary plodder along life's dusty highway, (who has toiled over many a hill that looked, from the distance, like a delicate fairy land, full of dim enchanting lights, and purple tender shadows, and found it only a repetition of the old rugged road, the rough scattered stones, and the laborious ascent, growing more and more toilsome from the increasing fatigue of the journey,) loses his interest in the newer stations, which become fewer and fewer, because he is disposed to hurry on to his ultimate destination, where he may finally rest from his labors. To him the past now becomes what the future once was. Memory takes the place of hope; the light now is all in the past; the cold shadows of evening rest upon life's future.

To one of these stages you have this evening attained. You have long been looking forward to this result, and now your hopes have expanded into fruition. You are just completing the term of your pupilage, just entering upon the great labor of life. The hill-top which seemed so difficult of access, along the road to which you have so sedulously toiled, has at length been reached, and you are now enjoying the fragrance and beauty of its accustomed flowers. Before you, in long perspective, stretches the untried future.

The haze veils the features of the landscape, but that haze only adds to the beauty of the distance, and the sun tints its borders with gold. The eye cannot detect the roughnesses and inequalities of the road; the rugged rocks and the sharp thorns are invisible, and imagination fills the future with visions of loveliness which few are so happy as to realize.

If I could lift up the veil from your hearts and read what is passing in your innermost thoughts, I should probably find very much the same anticipations in every bosom. You are thinking of your after career. Fortune is expected to lavish her favors upon you. Wealth and honors are only waiting for your acceptance. The trees hang laden with golden fruit, you have but to stretch out your hands and they are yours; and then, if your visions reach so far into the coming time, a quiet retreat is ready for you, in which old age may calmly await the inevitable hour, and sharing the serene coolness of the evening, may gently breathe out its last, as the soft breeze dies away in the silent air.

Now it is true that there are rewards in life, but they are the prize of unremitting toil. Golden fruit does crowd the bending boughs, but the trees grow high upon the cliffs and you must climb laboriously to reach them. The prizes of life are not to be had without the dust and heat of labor. Life is no lotos-eater's island, full of the sweets of indolence, no lubber-land, where the chickens run about ready trussed and cooked, and invite you to feast upon them. It is a race-course, in which many active runners are striving for the mastery, and you must be fleet as they are or fleeter. It is a battle-field, in which no one has leisure to pity a fallen foe. The shock of the charging squadrons overturns many a soldier, but his comrades cannot stop to pick him up; they rush on over his prostrate body, leaving him to struggle back into life alone, or to perish upon the plain. They are too busy to attend to him, they cannot even take time to avoid trampling upon him when he is down.

It is a busy and an active age in which you make your appearance upon the stage of life. Never before, in the history of our race, has there been so much mental stir. All over the world men are toiling to attain some desirable good, to add something to the general stock of knowledge. There is a peculiarity, however, in the character of this intellectual excitement. Heretofore, it has been manifested chiefly in the department of letters. In that old mental awakening which accompanied and immediately followed the Reformation, the activity was in the department of scholarship. Those were the days of enormous erudition. The human mind, shut out for so many ages from the master-pieces of classic authors, found it almost impossible to satiate its thirst for the beauty of those models of poetry, eloquence and philosophy. The knowledge of books and of languages became at once the aim of a man's life, and the measure of his intellectual powers. The great grammarians and critics, who towered as much above their successors as the giants of old are fabled to have exceeded the stature of ordinary men, left their impress upon their age and moulded its intellectual character. A life-time was spent in making commentaries upon an ancient author, or in settling the exact meaning and applicability of a Greek particle, and this sort of toil upon the machinery by which the knowledge of things is acquired, was taken taken for a true acquisition of real information. Up to the last moment, they earnestly and honestly pursued their labors, like the dying grammarian, who

> "With the throttling hands of Death at strife
>   Ground still at grammar;
> Still, through the rattle, parts of speech were rife.
>   While he could stammer,
> He settle *Hoti's* business—let it be!—
>   Properly based *Oun*—
> Gave us the doctrine of the enclitic *De*,
>   Dead from the waist down."

In our day, however, the course of things has changed. While there is no deficiency in learning, in the old acceptation of that term, the mere book, and the naked language are gradually falling back into the second rank. Facts are now the subject of study. The modern student grapples with the mysteries of nature, and strives to bring her forces under the control of intelligent mind. Those terrible potencies which the ancients regarded with such unaffected alarm, and which they symbolized in the dark and bloody side of the character of Cybele, the strange story of the Sphinx, and other myths, are now the servants of man. The expansive power of steam is subjected to the domination of mind. We see daily the dream of the old Arabian story-teller become an actual verity, surpassing far the wildest flight of even an oriental imagination. You all remember the story of the fisherman and the kettle, told in the Arabian Nights. You recollect how the fisherman went out early in the morning to his work, how he had bad luck, and abused the partiality of fortune which gave him no return for his toil. At last, he resolved before going home for the day, to cast his net once more into the sea. As he drags it up, he finds it heavy, congratulates himself on the prospect of a noble haul of fish, lugs it to the surface with no little trouble and finds in it only a copper kettle. Disgusted at his ill-luck, he is about to kick it back into the water, when he espies a seal upon the lid. He opens it. A great smoke rises from the kettle, towering high into the air, and gradually condenses, till the huge limbs and scowling brow of a monstrous genie threaten the terrified fisherman. I will not go over the minutiæ of the story, how the man overreached the monster, made a satisfactory bargain with him, and had him afterwards for his obsequious servant. I only say that civilization has her giant of the kettle, to do her bidding all over the world. You find him laboriously toiling everywhere; raising heavy burdens from deep mines, beating out refractory metals into still new weapons for the conquest of the brute forces of the universe; now hammering out the great iron machinery which is to impel the steamship across the stormy waters of the ocean, and again weaving the most delicate fabrics to adorn the graceful form of a modern beauty. Civilization also has tamed the lightning and taught it to carry her messages with a speed that outstrips the fleet pinions of time. The telegraphic dispatch which leaves here at this moment reaches St. Louis an hour in advance of that at which it is dated. It has left the sun behind it in its rapid flight. What was the magic carpet of that other Eastern story to such a feat as this?

Nor is it only in the attempt to make a practical application of cosmical forces that the modern student spends his time. He devotedly follows Nature through all her secret haunts. Armed with scalpel and microscope and test-glass, he pursues the footsteps of life through ocean, earth and air. No organism is too minute to escape, no desert too barren to lure, no distance too great to check his eager pursuit after knowledge. The secret recesses of the great deep are invaded, and their inner life laid bare to the inquisitive eye of modern research. Those mysteries which formerly only the speculation of philosophers and the imagination of poets ventured to approach, have now become the attraction of the museum and the amusement of the drawing-room. Alongside of the little elegancies of civilization, surrounded by mirrors, curtains and luxuries, the strange creatures of the sea indulge their appetites, and lead their "cool, sweet, silver lives, begirt with waves," unconscious of the prying eyes that watch their every motion. In the aquarium on the window-ledge, the sea-anemone unfolds its brilliant petals, the polypus grapples with his passing prey, the stickleback flashes from his secret recess, and the groves of seaweed spread their branching arms over the miniature rocks.

The hiding places of mother earth are also ransacked. Grim skeleton remains of strange forms that roamed through old carboniferous forests, of monsters that tempested the ancient ocean which deposited the Old Red Sandstone, of giant reptiles that devoured the fishes of a later era, have risen from the rocky tombs where they lay buried under an avalanche of centuries, and now confront us upon the shelves and in the cases of every geological museum. People talk about the antiquities of Egypt and India, and stand awed as by the visible presence of old Time himself, under the great lintels of Edfu, or the awful overhanging vaults of Elephanta. But in these exhumed remains of forgotton cycles, they confront a prodigious antiquity, before which that of the builders of the pyramids and the hewers out of the rock temples becomes a thing of yesterday. It is a step backward from out the circle of that measure of human generations which men call Time, into the wider sphere that surrounds it, and blends its distant boundaries with the illimitable cycles of Eternity. It is but natural that such gigantic remains should give birth to stupendous speculations, to daring hypotheses which attempt to unhinge all that we have considered fixed in history or sacred in religion. Doubtless, however, these bold innovations of modern scientific imaginations, like "the wild enormities of ancient beliefs," are destined to perish. After a longer or shorter struggle, a contest, it may be of years, or it may be of centuries, the ultimate triumph of Truth is sure to come; so that simple Faith and true Science can both afford to wait for that final reconciliation which is certainly approaching, and which only our imperfect perception of truth delays. Out of Chaos, Cosmos. It has been the rule for the visible universe; it is no less the rule for the world of speculation.

Not, only, however, does modern intellectual activity differ from ancient in the character of its pursuits; it is alike distinct in its spirit and inner life. Ancient scholarship was dogmatic and exclusive. The clerkly spirit of the middle ages, when an immense interval separated the learned man from the masses, was inherited by the early scholars of whom we have spoken. Learning established for itself a special aristocracy, receiving greater or less acknowledgment from the social aristocracy that environed it, but nevertheless as absolute and imperious as any that based its claims to consideration upon a long line of noble ancestry. The scholar considered himself a thing apart from the common man, a sort of Brahmin among a world of Pariahs, protected by a caste of Rajpoots. He resented, as something little short of sacrilege, any attempt on the part of the inferior rabble to meddle with those departments of human thought which he considered his peculiar province. He wrote and lived and thought, not for the great world, but for the little special circle that thought with him or cared for him. He had ever in his eye the "audience few but fitting," of his fellow-scholars and their noble protectors. So generation after generation saw a succession of men of brilliant parts, of great learning, of unwearying industry, toiling diligently in the same old round, and grinding sedulously the same old grain. The "verba magistri" still bound free thought, and most intellectual labor was spent upon exposition. Naturally, error was gravely and reverently handed down from age to age, as something too sacred to be trifled with.

Let us, however, do honor to these old students. Theirs was the first indication of vitality in the human mind awaking from its long sleep of ages, and if that vitality was not so active as it has become since intellect has been fully roused, we are not to find fault with it. They led the way, and thought gradually became freer and freer, till it reached its present liberty, which many might be inclined to call license.

The spirit of modern inquiry differs from all this. It is bold and free and democratic. No caste has the monopoly of its prerogatives. Whoever will may contribute his mite to the common stock, and derive the benefit of the whole accumulated knowledge of the race. Every man is now "the heir of all the ages." The Pierian spring is no longer on an inaccessible hill, guarded by divinities who allow their votaries only to bathe their lips in its divine waters, but it wells out by life's common highway, and any weary traveler may stop and drink.

Science is no longer a succession of commentaries upon the dicta of some authoritative teacher. The day when a man might sit in his study and excogitate a plan of the universe, which should be received by the world of letters as a true exposition of the mystery of nature, has long since passed by. Theory has shrunk into its proper limits, and no longer expands itself over the whole round of the sciences. The old notion that science was made up in part of facts, and in part of reasonings upon those facts, which made it a mixture of truth and speculation, each having equal weight, is now entertained by very few. Everything is brought to the infallible touchstone of experiment. The loftiest intellect cannot rrogate the right to dictate interpretations of natural phenomena, and the humblest votary of science, with a single discovered fact, has the recognized right to challenge the conclusions of the most renowned teacher. Indeed, the prominent characteristic of modern science is impartial candor. It busily accumulates facts, and every one of its votaries is taxed for his share.

Such, gentlemen, is the intellectual character of the age in which you are called upon to labor. I assume that, having received a liberal education, having acquired something from the stock of knowledge which has been accumulated by your predecessors, you are willing to make the only return in your power, a contribution to the common stock, to the full extent of your abilities and opportunities. The very nature of your calling allows you a certain amount of leisure. By a proper apportionment of your time, every one of you, even he who has the most to do, will be able to find abundant opportunity for the prosecution of some particular department of scientific inquiry. You have the control of your own time, to an extent never to be hoped for by those who devote themselves to the general practice of medicine. Out of this leisure and this education, we have a right to expect some satisfactory results. I feel satisfied that you will not all disappoint us.

The profession which you have chosen is one which has not fallen behind the general progress of the arts. Its advance has been most rapid within the past twenty years.

There are men now within the sound of my voice, who distinctly remember when dentistry occupied a position not a whit more respectable than the barber-surgery of old times. From this degradation it has been elevated to the rank of a co-ordinate branch of medicine, a department of an art which has no superior and few equals among all the occupations in which men engage; an art whose annals are radiant with a glory which no envy can dim; an art which has furnished to the world examples of heroic self-denial that may be admired and feebly imitated, but cannot be surpassed. How much of this elevation has been accomplished by the efforts of one of my colleagues, to whose instructions many successive classes of students have listened, and whose ripe experience is, we trust, destined to enlighten many more, I need not say. You all know how conspicuous have been the benefits he has conferred upon your art, and how very low it would sink this day, could what he has accomplished be blotted out from its history.

Among the elevating influences which he brought to bear none has been more important or more powerful than that of the College which has just conferred its honors upon you. I can speak freely upon this subject without laying myself open to any charge of want of modesty, for with the past of the College, at least, I have had no connexion, except that of an ardent well-wisher. For the future, it may become me to be silent. Of the past, I am certainly entitled to speak as a disinterested witness.

It cannot be denied that this College has made its mark upon the whole profession. It was the first school that was ever established for the instruction of students of dental surgery. Before its foundation, it was customary for a young man who wished to practice your art, to enter an office, or more commonly to wander about from dentist to dentist, to buy various secrets of trade; and men established in practice were not ashamed thus to peddle recipes and formulæ for work. The amount of information thus obtained was in most cases far from valuable, and this method of procuring it tended to lower the self-respect of all who engaged in such a system of barter. The respectability of a profession was therefore impossible for dentistry, so long as such a system prevailed. As the money must be put up before the secret was imparted, there was naturally a good deal of swindling on one side, and disappointment on the other, and the tendency of the whole business was to promote quackery of the meanest and most insufferable character. When, however, this College was established, an honorable and open method of acquiring valuable information was afforded to the student. The old jealously guarded secrets gradually came to light, and were disposed of according to their value. An interchange of views became possible among those who were really possessed of valuable information, an *esprit de corps* sprang up, and dentistry rose from a not very reputable trade to be a respectable and honorable profession.

Since then, other schools have arisen in our own country, and far be it from me to say any thing disparaging of them. Still, whatever credit is due to the collegiate system certainly belongs above all others to the school in which you have graduated, because it is the oldest of all, and because it has furnished the model upon which all the others have been constructed.

To your hands, gentlemen, and those of your fellow alumni, is committed the hard and honestly earned reputation of the Baltimore College. In keeping it up you will elevate yourselves as well as the standard of your profession. We ask you to prove to the world that the education professed to be given here is not mere idle talk to fill the columns of a circular, but a substantial reality. We expect you to show that your diplomas mean something, and that there is a very wide difference between you who have been regularly educated in your profession and those who, without previous study, have drifted into dentistry.

But, gentlemen, we have now sufficiently indulged in anticipations of the future. I have said that this is also a season for retrospect. Let us now turn our eyes toward the past. Alas! the "prospect is not good that way!"

I look round me and see one vacant chair. Among the accustomed group whom, year after year, I have met upon this platform, I miss one familiar face. The old foe of our race has been among us, and one of our number shall no more be seen among living men. The silent monarch of the tomb has beckoned with his skeleton finger, and our friend has risen and joined that mighty army which in one unbroken procession, daily, hourly, momentarily, marches into the "silent land." We cannot let him pass into those shadowy regions without a grateful acknowledgment of his past services, and an honest eulogy upon his many virtues. His long connection with this school, his high standing as a teacher, his well earned reputation throughout the country, make such a recognition eminently proper upon the present occasion.

My colleagues have selected me to render this last tribute of respect to the memory of our departed friend, and although the choice might have devolved upon one better qualified for the duty, yet in some respects the selection has a certain fitness. Years ago, when I commenced the study of medicine, Dr. Handy gave me my first lesson in practical anatomy; subsequently we became connected as professors in the same medical school, and from that time till the day of his death I was proud to call him friend, and had the privilege of that intimate association with him which gave me an opportunity of observing his rare qualities of mind and heart.

Your late Professor of Anatomy was born in Somerset County, on the Eastern Shore of Maryland, in the year 1812. He completed his preliminary education at the Classical Academy in Newark, Delaware, whence he went to West Point as a cadet. While there he was attacked with a severe indisposition, which totally incapacitated him for undergoing the rigid discipline of that institution, and compelled him to abandon the idea of serving his country in the profession of arms. For some time he was incapable of any exertion, mental or physical. Upon his recovery, he devoted himself assiduously to the study of medicine, and graduated with high distinction at the Washington Medical College of this city. Not long after he had received his diploma he was appointed Demonstrator of Anatomy in that school, and discharged the duties of that position with the zeal and energy that characterized him through life. In 1841 he was elected Professor of Anatomy in this College, and until a few weeks before his death, he continued his lectures. His teachings have enlightened many successive classes of students. You, gentlemen, have had the good fortune to sit under the sound of his voice; and upon you has fallen the great shadow of his loss. I need not, I am sure, recall to your memory his admirable qualities as a lecturer. You all know how faithful, how zealous, how untiring he was in his efforts to impart instruction; how completely he had the interests of the College at heart, how anxiously he labored to elevate the standard of your profession.

His long association with students led him to perceive more clearly than most men their particular wants. He was convinced that the ordinary method of instruction in Anatomy left much to be desired. He thought that the habit of treating one set of organs by themselves, without reference to others immediately connected with them was wrong, and tended to confuse the student and to deprive the study of much of the interest which ought to attach to it. Dr. Handy altered, and, in my opinion, greatly amended the plan of teaching this science. He studied the various organs in their natural relations. In this way the student obtained a comprehensive view of the whole organization of a part; the deep meaning of its minute structure became clear; anatomy was vitalized for him, and assumed the attractiveness of physiology. In 1854 he published a treatise on anatomy, arranged upon this plan, which has become a text-book in all the dental colleges of this country.

Of the character of our deceased friend, it is difficult to speak in terms of moderate eulogy. He was emphatically the "*justum et tenacem propositi virum*" whom Horace praises so highly. It would be difficult to find anywhere a man so rigorously and scrupulously just. In his whole career, not only did never a spot sully the purity of his reputation, but no one who knew him ever dreamed that he could for a moment even design an unjust or dishonorable action. His was that inflexible integrity which has been so lauded among the ancient Romans, and is so rarely found in our degenerate days. His fixed and unalterable determination was always to do right, and his whole life was characterized by a high morality which was incapable of accommodation to circumstances. He never suffered his own convenience or inclinations to interfere with what he considered right.

In deciding upon any particular line of action, he was extremely cautious. He weighed well all the arguments upon either side of the question, deliberated calmly, and while engaged in determining the point, was always open to conviction; but having once made up his mind, his decision was final and irrevocable. No argument, no entreaty, no threats could move him from his purpose. Intimidation signally failed with him, for that brave heart never quailed before mortal man.

Severe, however, as were his principles, in social intercourse he was the most affable and amiable of men. He was studiously considerate of the feelings of others, and rarely allowed an expression to escape him which could wound the most sensitive nature. Even when roused to honest indignation by marked injustice, he gave expression to his feelings in measured language, and did not suffer the excitement of the occasion to carry his expressions beyond the literal truth. In his intercourse with his brother practitioners, he was not only courteous, but most scrupulous in observing all the little points of medical etiquette. Indeed, tenacious as he naturally was of his own rights, I have known him more than once to sacrifice them rather than incur the suspicion of an inclination to violate those of others. His self-respect led him to demand of others the same even-handed justice which he accorded to them, and if necessary, he did not hesitate to extort it.

He was also a man of rare purity of heart. Modest as a girl, his long intercourse with the world had left him singularly uncontaminated. In the midst of an active practice, he preserved a chastity of life and character which many a cloistered monk, spending his weary years in mortifying the flesh, sighs for in vain.

Intellectually, his predominant trait was an ardent love of truth. He subjected all theories to the infallible test of facts, severely analyzed them, and impartially rejected whatever could not bear this *experimentum crucis.* The nature of his mind was such that he demanded the most rigorous demonstration, the most absolute certainty, and could be satisfied with nothing else.

Upon the particulars of his last illness, I do not propose to dwell. It has too recently passed to enable us to speak of it with composure. You all remember the long anxiety with which you heard of its daily changes; your doubts, your trembling hopes, your foreboding fears; while over him

> "Triumphant Death his dart
> Shook, but delayed to strike."

His illness was, as you all know, a painful one, painful to himself and to his friends, on account of the intellectual darkness which from time to time descended upon him. From the beginning, he himself was assured of its fatal termination; but for him death had no terrors. Across the gathering gloom of the present, his eye of faith saw clearly the everlasting brightness of the future, expanding as he neared it, increasing ever from glory to glory, till the luminous heavens received him, and set the bright barrier of stars between him and us. For us, who have watched his spirit lessening up the blue depths, remains the grief, and the longing and the emptiness of heart—for him, the blessedness of the eternal reward. We sorrow not, therefore, as those without hope, but still our wailings to listen to the voice from heaven, saying, "Blessed are the dead who die in the Lord."

